# Supplementary material for: COVID-19 Infections Still Occur: How Do Pregnant and Non-Pregnant Individuals Compare? A Study from the Canadian Mother–Child Initiative on Drug Safety in Pregnancy (CAMCCO)
Source: Int J Environ Res Public Health. 2025 Nov 20;22(11):1756. doi: 10.3390/ijerph22111756 (PMC12652618; doi:10.3390/ijerph22111756)
Supplement: Supplementary file 1 [file ijerph-22-01756-s001.zip › ijerph-3950376-supplementary.docx]

**SUPPLEMENTAL FILES**

**COVID-19 infections still occur: How do pregnant and non-pregnant individuals compare?**

**A study from the Canadian Mother-Child Initiative on Drug Safety in Pregnancy (CAMCCO)**

Bérard A,^1.,2,3^ Sheehy O,^1^ Kaul P,^4^ Eltonsy S,^5^ Walker M,^6,7^ Hawken S,^6,7^ Bernatsky S,^8^ Pugliese M,^6,7^ Barrett O,^4^ Savu A,^4^ Dragan R^5^

Table S1. ATC-level 2

| **ATC-level 2 codes of medication groups of special relevance to COVID-19 (WHO LIST (10))** |
| --- |
| Drugs used in diabetes (A10) |
| Antithrombotic agents (B01) |
| Antihypertensives (C02, C03, C04, C07, C08 and/or C09) |
| Corticoisteroids (H02) |
| Antibacterials (J01) |
| Antimycotics (J02) |
| Antimycobacterials (J04) |
| Antivirals (J05) |
| Immune sera and globulins (J06) |
| Vaccinations (J07) |
| Antineoplastic agents (L01) |
| Immunostimulants (L03) |
| Immunosuppressants (L04) |
| Anti-inflammatory drugs (M01) |
| Antigout preparations (M04) |
| Analgesics (N02) |
| Psycholeptics (N05) |
| Psychoanaleptics (N06) |
| Antiprotozoals (P01) |
| Antihelminthics (P02) |
| Nasal preparations (R01) |
| Medicines for obstructive airway disease (R03) |
| Cough and cold medications (R05) |

**Table S2. ICD-9-CM and ICD-10-CM codes for spontaneous abortion**

| **ICD-9-CM**  **Diagnostic codes** | **Description** |
| --- | --- |
| 632 | Missed abortion |
| 634 | Spontaneous abortion |
| 634.0 | Spontaneous abortion complicated by genital tract and pelvic infection |
| 634.00 | Spontaneous abortion, complicated by genital tract and pelvic infection, unspecified |
| 634.01 | Spontaneous abortion, complicated by genital tract and pelvic infection, incomplete |
| 634.02 | Spontaneous abortion, complicated by genital tract and pelvic infection, complete |
| 634.1 | Spontaneous abortion complicated by delayed or excessive hemorrhage |
| 634.11 | Spontaneous abortion, complicated by delayed or excessive hemorrhage, incomplete |
| 634.12 | Spontaneous abortion, complicated by delayed or excessive hemorrhage, complete |
| 634.2 | Spontaneous abortion complicated by damage to pelvic organs or tissues |
| 634.20 | Spontaneous abortion, complicated by damage to pelvic organs or tissues, unspecified |
| 634.21 | Spontaneous abortion, complicated by damage to pelvic organs or tissues, incomplete |
| 634.22 | Spontaneous abortion, complicated by damage to pelvic organs or tissues, complete |
| 634.3 | Spontaneous abortion complicated by renal failure |
| 634.31 | Spontaneous abortion, complicated by renal failure, incomplete |
| 634.32 | Spontaneous abortion, complicated by renal failure, complete |
| 633.1 | Tubal abortion |
| 634.4 | Spontaneous abortion complicated by metabolic disorder |
| 634.40 | Spontaneous abortion, complicated by metabolic disorder, unspecified |
| 630 | Hydatidiform mole |
| 631 | Other abnormal product of conception |

| **ICD-10-CM**  **Diagnostic codes** | **Description** |
| --- | --- |
| O01 | Hydatidiform mole |
| O020 | Blighted ovum and nonhydatidiform mole |
| O02.1 | Missed abortion |
| O02.8 | Other specified abnormal products of conception |
| O02.9 | Abnormal product of conception, unspecified |
| O03 | Spontaneous abortion |
| O03.1 | Spontaneous abortion, incomplete, complicated by delayed or excessive haemorrhage |
| O03.2 | Spontaneous abortion, incomplete, complicated by embolism |
| O03.2 | Air embolism following incomplete spontaneous abortion |
| O03.2 | Amniotic fluid embolism following incomplete spontaneous abortion |
| O03.2 | Blood-clot embolism following incomplete spontaneous abortion |
| O03.2 | Fat embolism following incomplete spontaneous abortion |
| O03.2 | Pulmonary embolism following incomplete spontaneous abortion |
| O03.2 | Pyemic embolism following incomplete spontaneous abortion |
| O03.2 | Septic or septicopyemic embolism following incomplete spontaneous abortion |
| O03.2 | Soap embolism following incomplete spontaneous abortion |
| O03.2 | Spontaneous abortion, incomplete, complicated by embolism |
| O03.33 | Metabolic disorder following incomplete spontaneous abortion |
| O03.4 | Spontaneous abortion, incomplete, without complication |
| O03.9 | Miscarriage NOS |
| **Procedure codes** | Quebec codes and each province must find their equivalent codes (AL, MA, ON) |
| Incomplete abortion : | |
| 06900 | by menstrual extraction |
| 06906 | by curettage |

**Table S3. ICD-9-CM and ICD-10-CM diagnostic codes for eclampsia and preeclampsia**

| **ICD-9-CM**  **Diagnostic codes** | **Description** |
| --- | --- |
| Preeclampsia | |
| 642 | Hypertension complicating pregnancy, childbirth, and the  puerperium |
| 642.1 | Hypertension secondary to renal disease, complicating pregnancy,  childbirth, and the puerperium |
| 642.3 | Transient hypertension of pregnancy |
| 642.4 | Pre-eclampsia (mild) or unspecified |
| 642.5 | Severe pre-eclampsia |
| 642.7 | with pre-existing hypertension |
| 642.7 | superimposed on pre-existing hypertensive disease |
| 642.9 | Unspecified hypertension complicating pregnancy, childbirth, or  the puerperium |
| Eclampsia | |
| 642.6 | during pregnancy, childbirth, or puerperium |
| 642.7 | with pre-existing hypertension |
| 760.0 | affecting fetus or newborn |
| 646.20 | Unspecified renal disease in pregnancy, without mention of  hypertension, unspecified as to episode of care or not applicable |
| 646.23 | Unspecified renal disease in pregnancy, without mention of  hypertension, antepartum condition or complication |
| **ICD-10-CM**  **Diagnostic codes** | **Description** |
| Preeclampsia | |
| O10.0 | Pre-existing essential hypertension complicating pregnancy,  childbirth and the puerperium |

| O12 | Gestational [pregnancy-induced] oedema and proteinuria without  hypertension |
| --- | --- |
| O12.1 | Gestational proteinuria |
| O13 | Gestational hypertension NOS |
| O13 | Transient hypertension of pregnancy |
| O14 | Pre-eclampsia |
| O14.1 | Severe pre-eclampsia |
| O14.2 | HELLP syndrome |
| O14.20 | HELLP syndrome (HELLP), unspecified trimester |
| O14.24 | HELLP syndrome, complicating childbirth |
| O14.9 | Pre-eclampsia, unspecified |
| O14.90 | Unspecified pre-eclampsia, unspecified trimester |
| Eclampsia | |
| O15 | Eclampsia |
| O15.0 | Eclampsia complicating pregnancy |
| O15.00 | Unspecified trimester |
| O15.02 | Second trimester |
| O15.03 | Third trimester |
| O15.1 | Eclampsia complicating labor |
| O15.2 | Eclampsia complicating the puerperium |
| O15.9 | Eclampsia, unspecified as to time period |

**Table S4. ICD-9-CM and ICD-10-CM codes for Gestational diabetes**

| **ICD-9-CM**  **Diagnostic codes** | **Description** |
| --- | --- |
| 648.0 | Diabetes mellitus complicating pregnancy, childbirth, or the  puerperium |
| 648.00 | Diabetes mellitus of mother, complicating pregnancy, childbirth, or  the puerperium, unspecified as to episode of care or not applicable |
| 648.01 | Diabetes mellitus of mother, complicating pregnancy, childbirth, or  the puerperium, delivered, with or without mention of antepartum condition |
| 648.02 | Diabetes mellitus of mother, complicating pregnancy, childbirth, or  the puerperium, delivered, with mention of postpartum complication |
| 648.03 | Diabetes mellitus of mother, complicating pregnancy, childbirth, or  the puerperium, antepartum condition or complication |
| 648.04 | Diabetes mellitus of mother, complicating pregnancy, childbirth, or  the puerperium, postpartum condition or complication |
| 648.8 | Abnormal glucose tolerance of mother, complicating pregnancy,  childbirth, or the puerperium |
| 648.80 | Abnormal glucose tolerance of mother, unspecified as to episode of  care or not applicable |
| 648.81 | Abnormal glucose tolerance of mother, delivered, with or without  mention of antepartum condition |
| 648.82 | Abnormal glucose tolerance of mother, delivered, with mention of  postpartum complication |
| 648.83 | Abnormal glucose tolerance of mother, antepartum condition or  complication |
| 648.84 | Abnormal glucose tolerance of mother, postpartum condition or  complication |

| **ICD-10-CM**  **Diagnostic codes** | **Description** |
| --- | --- |
| E08-E13 | Diabetes mellitus (E08-E13) |
| E10-E14.9 | Diabetes mellitus |
| E14 | Unspecified diabetes mellitus |
| E74.9 | Disorder of carbohydrate metabolism, unspecified |
| O24 | Diabetes mellitus in pregnancy |
| O24 | Diabetes in pregnancy, childbirth, and the puerperium |
| O24.4 | Diabetes mellitus arising in pregnancy |
| O24.41 | Gestational diabetes mellitus in pregnancy |
| O24.410 | Gestational diabetes mellitus in pregnancy, diet controlled |
| O24.414 | Gestational diabetes mellitus in pregnancy, insulin controlled |
| O24.419 | Gestational diabetes mellitus in pregnancy, unspecified control |
| O24.42 | Gestational diabetes mellitus in childbirth |
| O24.420 | Gestational diabetes mellitus in childbirth, diet controlled |
| O24.424 | Gestational diabetes mellitus in childbirth, insulin controlled |
| O24.429 | Gestational diabetes mellitus in childbirth, unspecified control |
| O24.43 | Gestational diabetes mellitus in the puerperium |
| O24.430 | Gestational diabetes mellitus in the puerperium, diet controlled |
| O24.434 | Gestational diabetes mellitus in the puerperium, insulin controlled |
| O24.439 | Gestational diabetes mellitus in the puerperium, unspecified control |
| O24.9 | Diabetes mellitus in pregnancy, unspecified |
| O24.9 | Unsp diabetes in pregnancy, childbirth and the puerperium |
| O24.91 | Unspecified diabetes mellitus in pregnancy |
| O24.919 | Unspecified diabetes mellitus in pregnancy, unspecified trimester |
| R73.9 | Hyperglycaemia, unspecified |
| R81 | Glycosuria |

**Table S5. ICD-9-CM and ICD-10-CM codes for Termination of Pregnancy for Fetal Anomaly (TOPFA)**

| **ICD-9-CM**  **Diagnostic codes** | **Description** |
| --- | --- |
| 655.91 | Unspecified suspected fetal abnormality, affecting management of mother, delivered, with or without mention of antepartum condition |
| 655.93 | Unspecified suspected fetal abnormality, affecting management of mother, antepartum condition or complication |
| **ICD-10-CM**  **Diagnostic codes** | **Description** |
| O35 | Maternal care for known or suspected fetal abnormality and damage |

**Table S6. CCI codes for caesarean**

| **CCI codes** | **Description** |
| --- | --- |
| 5MD60AA | C section |
| 5MD60CB | Cesarean hysterectomy w vacuum and forceps |
| 5MD60CC | Classic C section w vacuum & forceps |
| 5MD60CD | Extraperitoneal C section w vacuum & forceps |
| 5MD60CE | Inverted T C section w vacuum & forceps |
| 5MD60CF | Low segment C section w vacuum & forceps |
| 5MD60CG | C section NEC w vacuum & forceps |
| 5MD60JW | Low segment C section w forceps |
| 5MD60JX | Low segment C section w vacuum |
| 5MD60JY | Classical C section |
| 5MD60JZ | Classical C section w forceps |
| 5MD60KA | Classical C section w vacuum |
| 5MD60KB | Extraperitoneal C section |

| 5MD60KC | Extraperitoneal C section w forceps |
| --- | --- |
| 5MD60KD | Extraperitoneal C section w vacuum |
| 5MD60KE | Cesarean hysterectomy |
| 5MD60KF | Cesarean laparotomy (abd preg) |
| 5MD60KG | Inverted T C section |
| 5MD60KT | C section other type NEC |
| 5MD60RA | Inverted T C section w forceps |
| 5MD60RB | Inverted T C section w vacuum |
| 5MD60RC | Cesarean hysterectomy w forceps |
| 5MD60RD | Cesarean hysterectomy w vacuum |
| 5MD60RE | Cesarean laparotomy (abd preg) w forceps |
| 5MD60RF | Cesarean laparotomy (abd preg) w vacuum |
| 5MD60RG | Other type C section NEC w forceps |
| 5MD60RH | Other type C section NEC w vacuum |

**Table S7. ICD-9-CM and ICD-10-CM diagnostic codes for preterm birth**

| **ICD-9-CM**  **Diagnostic codes** | **Description** |
| --- | --- |
| 765.1 | Other preterm infants |
| 765.10 | Other preterm infants, unspecified [weight] |
| 765.11 | Other preterm infants, less than 500 grams |
| 765.12 | Other preterm infants, 500-749 grams |
| 765.13 | Other preterm infants, 750-999 grams |
| 765.14 | Other preterm infants, 1,000-1,249 grams |
| 765.15 | Other preterm infants, 1,250-1,499 grams |
| 765.16 | Other preterm infants, 1,500-1,749 grams |
| 765.17 | Other preterm infants, 1,750-1,999 grams |
| 765.18 | Other preterm infants, 2,000-2,499 grams |
| 765.19 | Other preterm infants, 2,500 grams and over |
| 765.21 | Less than 24 completed weeks of gestation |
| 765.22 | 24 completed weeks of gestation |
| 765.23 | 25-26 completed weeks of gestation |
| 765.24 | 27-28 completed weeks of gestation |
| 765.25 | 29-30 completed weeks of gestation |
| 765.26 | 31-32 completed weeks of gestation |
| 765.27 | 33-34 completed weeks of gestation |
| 765.28 | 35-36 completed weeks of gestation |

| 774.2 | Neonatal jaundice associated with preterm delivery |
| --- | --- |
| 776.6 | Anemia of prematurity |
| **ICD-10-CM**  **Diagnostic codes** | **Description** |
| O60.10 | Preterm labor with preterm delivery, unspecified trimester, not  applicable or unspecified |
| O60.10X0 | Preterm labor with preterm delivery, unspecified trimester, not  applicable or unspecified |
| O60.13 | Preterm labor second trimester with preterm delivery third trimester |
| O60.14 | Preterm labor third trimester with preterm delivery third trimester |
| P07.3 | Preterm [premature] newborn [other] |
| P07.3 | Prematurity NOS |

**Table S8. ICD-9-CM and ICD-10-CM diagnostic codes for stillbirth**

| **ICD-10-CM**  **Diagnostic codes** | **Description** |
| --- | --- |
| Z37.1 | Single stillbirth |
| Z37.3 | Twins, one liveborn and one stillborn |
| Z37.4 | Twins, both stillborn |
| Z37.60 | Multiple births, unspecified, some liveborn |
| Z37.61 | Triplets, some liveborn |
| Z37.62 | Quadruplets, some liveborn |
| Z37.63 | Quintuplets, some liveborn |
| Z37.64 | Sextuplets, some liveborn |
| Z37.69 | Other multiple births, some liveborn |
| Z37.7 | Other multiple births, all stillborn |

| **ICD-10-CM**  **Diagnostic codes** | **Description** |
| --- | --- |
| Z37.1 | Single stillbirth |
| Z37.3 | Twins, one liveborn and one stillborn |
| Z37.4 | Twins, both stillborn |
| Z37.60 | Multiple births, unspecified, some liveborn |
| Z37.61 | Triplets, some liveborn |
| Z37.62 | Quadruplets, some liveborn |
| Z37.63 | Quintuplets, some liveborn |
| Z37.64 | Sextuplets, some liveborn |
| Z37.69 | Other multiple births, some liveborn |
| Z37.7 | Other multiple births, all stillborn |

**Table S9. ICD-9-CM and ICD-10-CM diagnostic codes for LBW**

| **ICD-9-CM**  **Diagnostic codes** | **Description** |
| --- | --- |
| 764.0 | "Light-for-dates" without mention of fetal malnutrition |
| 764.05 | "Light-for-dates" without mention of fetal malnutrition, 1,250- 1,499  grams |
| 764.06 | "Light-for-dates" without mention of fetal malnutrition, 1,500- 1,749  grams |
| 764.08 | "Light-for-dates" without mention of fetal malnutrition, 2,000- 2,499  grams |
| 764.9 | Fetal growth retardation, unspecified |
| 764.90 | Fetal growth retardation, unspecified, unspecified [weight] |
| 765.01 | Extreme immaturity, less than 500 grams |
| 765.02 | Extreme immaturity, 500-749 grams |
| 765.03 | Extreme immaturity, 750-999 grams |

| 765.04 | Extreme immaturity, 1,000-1,249 grams |
| --- | --- |
| 765.05 | Extreme immaturity, 1,250-1,499 grams |
| 765.06 | Extreme immaturity, 1,500-1,749 grams |
| 765.07 | Extreme immaturity, 1,750-1,999 grams |
| 765.08 | Extreme immaturity, 2,000-2,499 grams |
| 765.11 | Other preterm infants, less than 500 grams |
| 765.12 | Other preterm infants, 500-749 grams |
| 765.13 | Other preterm infants, 750-999 grams |
| 765.14 | Other preterm infants, 1,000-1,249 grams |
| 765.15 | Other preterm infants, 1,250-1,499 |
| 765.16 | Other preterm infants, 1,500-1,749 grams |
| 765.17 | Other preterm infants, 1,750-1,999 grams |
| 765.18 | Other preterm infants, 2,000-2,499 grams |
| **ICD-10-CM**  **Diagnostic codes** | **Description** |
| P05.9 | Slow fetal growth, unspecified |
| P07 | Disorders related to short gestation and low birth weight, not  elsewhere classified |
| P07.0 | Extremely low birth weight newborn |
| P07.0 | Newborn birth weight 999 g. or less |
| P07.00 | Extremely low birth weight newborn, unspecified weight |
| P07.01 | Extremely low birth weight newborn, less than 500 grams |
| P07.02 | Extremely low birth weight newborn, 500-749 grams |
| P07.03 | Extremely low birth weight newborn, 750-999 grams |
| P07.1 | Other low birth weight |
| P07.1 | Newborn birth weight 1000-2499 g. |
| P07.10 | Other low birth weight newborn, unspecified weight |
| P07.14 | Other low birth weight newborn, 1000-1249 grams |
| P07.15 | Other low birth weight newborn, 1250-1499 grams |

| P07.16 | Other low birth weight newborn, 1500-1749 grams |
| --- | --- |
| P07.17 | Other low birth weight newborn, 1750-1999 grams |
| P07.18 | Other low birth weight newborn, 2000-2499 grams |
| R63.6 | Underweight |

**Table S10. ICD-9-CM and ICD-10-CM diagnostic codes for SGA/IURG**

| **ICD-9-CM**  **Diagnostic codes** | **Description** |
| --- | --- |
| 656.5 | Poor fetal growth affecting management of mother |
| 656.50 | Poor fetal growth, affecting management of mother, unspecified as to  episode of care or not applicable |
| 658.0 | Oligohydramnios |
| 658.00 | Oligohydramnios, unspecified as to episode of care or not applicable |
| 658.01 | Oligohydramnios, delivered, with or without mention of antepartum  condition |
| 764 | Slow fetal growth and fetal malnutrition |
| 764.9 | Fetal growth retardation, unspecified |
| 764.90 | Fetal growth retardation, unspecified, unspecified [weight] |
| 764.91 | Fetal growth retardation, unspecified, less than 500 grams |
| 764.92 | Fetal growth retardation, unspecified, 500-749 grams |
| 764.93 | Fetal growth retardation, unspecified, 750-999 grams |
| 764.94 | Fetal growth retardation, unspecified, 1,000-1,249 grams |
| 764.95 | Fetal growth retardation, unspecified, 1,250-1,499 grams |
| 764.96 | Fetal growth retardation, unspecified, 1,500-1,749 grams |
| 764.97 | Fetal growth retardation, unspecified, 1,750-1,999 grams |
| 764.98 | Fetal growth retardation, unspecified, 2,000-2,499 grams |
| 764.99 | Fetal growth retardation, unspecified, 2,500 grams and over |

| **ICD-10-CM**  **Diagnostic codes** | **Description** |
| --- | --- |
| O41.0 | Oligohydramnios |
| O41.00 | Oligohydramnios, unspecified trimester |
| O41.00X0 | Oligohydramnios, unspecified trimester, not applicable or unspecified |
| O43.89 | Placental dysfunction |
| P05 | Slow fetal growth and fetal malnutrition |
| P05.0 | Newborn light for gestational age |

**Table S11. ICD-9-CM and ICD-10-CM diagnostic codes for major congenital anomalies**

| **Organ system** | **ICD-9-CM**  **Diagnostic codes** | **ICD-10-CM**  **diagnostic codes** |
| --- | --- | --- |
| Nervous system | 740.0-742.9 | Q00.0-Q07.9 |
| Eye, ear, face and neck | 743.0-744.9  *Exclusions: 743.6, 743.8, and 744.1-744.9* | Q10.0-Q18.9  *Exclusions: Q10.0-Q10.6, Q13.0, Q13.2, Q13.5, Q15.8, Q17.0-Q17.5, Q17.8, Q17.9, and Q18.0-Q18.9* |
| Circulatory system | 745.0-747.9  *Exclusions: 747.0 and 747.5* | Q20.0-Q20.9  *Exclusions: Q25.0 and Q27.0* |
| Respiratory system | 748.0-748.9  *Exclusions: 748.2 and 748.3* | Q30.0-Q34.9  *Exclusions: Q30.2, Q30.8, Q31-Q32, and Q33.1* |
| Orofacial clefts | 749.0-749.2 | Q35.0-Q37.9  *Exclusions: Q35.7* |
| Digestive system | 750.0-751.9  *Exclusions: 750.0-750.2, 750.5, 750.6,*  *751.1, and 751.5* | Q38.0-Q45.9  *Exclusions: Q38.1-Q38.6, Q40.0, Q40.1, Q43.0, Q43.4-Q43.9* |

| Genital organs | 752.0-752.9  *Exclusions: 752.4, 752.5, and 752.8* | Q50.0-Q56.9  *Exclusions: Q52.2-Q52.8, Q53, Q54.4, Q55.1, Q55.2, Q55.6, Q55.8, and Q55.9* |
| --- | --- | --- |
| Urinary system | 753.0-753.9  *Exclusion: 753.6* | Q60.0-Q64.9  *Exclusions: Q61.0, Q62.7, Q63.3, Q64.2, and Q64.3* |
| Musculoskeletal system | 754.0-756.9  *Exclusions: 754.0, 754.1, 754.7,* 754.8,  *756.0, and 756.2* | Q65.0-Q79.9  *Exclusions: Q65.3-Q65.6, Q66.2, Q66.3, Q66.5-Q66.9, Q67.0-Q674, Q67.6-Q67.8,*  *Q68.0, Q68.1, Q68.3-Q68.8, Q70.3, Q74.1,*  *Q75.0, Q75.2, Q75.3, Q75.8, Q76.0, Q76.5, Q79.5, and Q79.8* |
| Integument | 757.0-757.9  *Exclusions: 757.2-757,6, and 757.8* | Q80.0-Q84.9  *Exclusions: Q81, Q82.1-Q82.8, Q83.2, Q83.3, Q83.8, Q84.1-Q84.6, and Q84.8* |
| Chromosomal | 758.0-758.8  *Exclusion: 758.4* | Q90.0-Q99.2  Exclusions: Q95.0 and Q95.1 |
| Other | 758.9-759.8 | Q85.0-Q89.9 and Q99.9  Exclusion: Q89.9 |

**Table S12. ICD-9-CM and ICD-10-CM diagnostic codes for medical condition and medicinal products ATC codes for the identification of the covariates of interest**

| **At-risk medical conditions (ICD-9 and ICD-10 codes)** | **Medicinal product proxy(ies) ATC codes** | |
| --- | --- | --- |
| **Cardiovascular incl. blood** | | |
| Cardiovascular disease /  Serious heart conditions including heart failure / Coronary artery disease / Cardiomyopathy  ***Circulatory system diseases***  ICD-9 codes : 413.9; 410.11;  410.91; 414.01; 414.00; 412; 414.4;  and 414.9  ICD-10 codes: I20.9; I21.09; I21.3; I25.10; I25.2; I25.84; I25.9  ***Ischemic heart disease***  ICD-9 code : 427.31; 428.00;  429.20; 433.10; 433.11; 434.91;  437.00; 437.10; and 433.9  ICD-10 code: I21.09; I25.2; I20.9;  and I25.10 | Antiarrhythmics, class 1 and 111 (C01B) | C01BD01 - Amiodarone C01BD02 - Bretylium tosilate C01BA03 - Disopyramide C01BD07 - Dronedarone C01BC04 – Flecainide C01BD05 - Ibutilide C01BB01 - Lidocaine C01BB02 - Mexiletine C01BA02 - Procainamide C01BC03 - Propafenone C01BA01 – Quinidine C01BB03 - Tocainide C01BG11 - Vernakalant |
|  | Cardiac stimulants excl. cardiac glycosides (C01C) | C01CE01 - Amirinone C01CA07 – Dobutamine C01CA04 – Dopamine C01CA26 – Ephedrine C01CA24 – Epinephrine C01CA02 – Isoprenaline C01CA11 – Mephentermine C01CA10 – Methoxamine C01CA17 – Midodrine C01CE02 – Milrinone C01CA03 – Norepinephrine C01CA06 - Phenylephrine |
|  | Vasodilators used in cardiac diseases (C01D) | C01DA02 – Glyceryl trinitrate C01DA08 – Isosorbide dinitrate C01DA14 – Isosorbide mononitrate C01DX19 – Nesiritide  C01DA05 – Pentaerithrityl tetranitrate |
|  | Other cardiac preparations (C01E) | C01EB10 – Adenosine C01EA01 – Alprostadil C01EB03 – Indometacin C01EB17 – Ivabradine C01EB18 – Ranolazine C01EB09 - Ubidecarenone |
|  | Antithrombotic agents (B01A) | B01AC13 – Abciximab B01AA07 – Acenocoumarol  B01AC06 – Acetylsalicyclic acid B01AD02 – Alteplase  B01AD09 – Ancrod |

| **At-risk medical conditions (ICD-9 and ICD-10 codes)** | **Medicinal product proxy(ies) ATC codes** | |
| --- | --- | --- |
|  |  | B01AD03 – Anistreplase B01AB02 – Antithrombin B01AF02 – Apixaban B01AE03 – Argatroban B01AE06 – Bivalirudin B01AX07 – Caplacizumab B01AC04 – Clopidogrel  B01AE07 – Dabigatran etexilate B01AB04 – Dalteparin  B01AB09 – Danaparoid B01AX01 – Defibrotide  B01AB51 – Heparin, combinations B01AC30 – Combinations B01AD10 – Drotrecogin alfa B01AF03 – Edoxaban  B01AB05 – Enoxaparin B01AC09 – Epoprostenol B01AC16 – Eptifibatide B01AX05 – Fondaparinux B01AB01 - Heparin  B01AB51 – Heparin, combinations B01AE02 – Lepirudin  B01AB06 – Nadroparin B01AC22 – Prasugrel B01AD07 – Reteplase B01AF01 – Rivaroxaban B01AC27 – Selexipag B01AD01 – Streptokinase B01AD11 – Tenecteplase B01AC24 – Ticagrelor B01AC05 – Ticlopidine B01AB10 – Tinzaparin B01AC17 – Tirofiban B01AC21 – Treprostinil B01AD04 – Urokinase B01AC26 – Vorapaxar B01AA03 – Warfarin |
| ***Hypertension***  ICD-9 codes: 401-405; 642 and  796.2  ICD-10 codes: I10-I13; I15; O10- O16 |  | C07AB04 - Acebutolol C09XA02 - Aliskirene C09XA52 - Aliskirene/HCTZ C03DB01 - Amiloride C03EA01 - Amiloride/HCTZ C08CA01 - Amlodipine  C10BX03 - Amlodipine/Atorvastatine C09BB04 - Amlodipine/Perindopril C09DB04 - Amlodipine/Termisartan C07AB03 - Atenolol  C07CB03 - Atenolol/Chlorthalidone C09CA09 - Azilsartan medoxomil |

| **At-risk medical conditions (ICD-9 and ICD-10 codes)** | **Medicinal product proxy(ies) ATC codes** | |
| --- | --- | --- |
|  |  | C09DA09 - Azilsartan/Chlorthalidone C09AA07 - Benazepril  C03AA01 - Bendroflumethiazide C07AB07 - Bisoprolol  C09CA06 - Candesartan C09DA06 - Candesartan/HCTZ C09AA01 - Captopril C07AG02 - Carvedilol C03AA03 - Chlorothiazide C03BA04 - Chlorthalidone C09AA08 - Cilazapril  C09BA08 - Cilazapril/HCTZ C08CA16 - Clevidipine C02AC01 - Clonidine C08DB01 - Diltiazem C02CA04 - Doxazosin C09AA02 - Enalapril C09BA02 - Enalapril/HCTZ C03DA04 - Eperenone C09CA02 - Eprosartan C09DA02 - Eprosartan/HCTZ C07AB09 - Esmolol C03CC01 - Ethacryique C08CA02 - Felodipine C09AA09 - Fosinopril C03CA01 - Furosemide C02DB02 - Hydralazine  C03AA03 - Hydrochlorothiazide C09DA04 - Irbesartan/HCTZ C07AG01 - Labetalol  C09AA03 - Lisinopril C09BA03 - Lisinopril/HCTZ C09CA01 - Losartan C09DA01 - Losartan/HCTZ C03BA08 - Metolazone C07AB02 - Metoprolol C02AB02 - Methyldopa C02LB01 - Methyldopa/HCTZ C02DC01 - Minoxidil C07AA12 - Nadolol  C07BA12 - Nadolol/Bendroflumethiazide C08CA04 - Nicardipine  C08CA05 - Nifedipine C08CA06 - Nimodipine  C09CA08 - Olmesartan medoxomil C09DA08 - Olmesartan medoxomil/HCTZ C07AA02 - Oxprenolol  C09AA04 - Perindopril  C09BA04 - Perindopril/Indapamide C07AA03 - Pindolol |

| **At-risk medical conditions (ICD-9 and ICD-10 codes)** | **Medicinal product proxy(ies) ATC codes** | |
| --- | --- | --- |
|  |  | C07CA03 - Pindolol/HCTZ C02CA01 - Prazosin C07AA05 - Propranolol C09AA06 - Quinapril C09BA06 - Quinapril/HCTZ C09AA05 - Ramipril  C09BB05 - Ramipril/Felodipine C09BA05 - Ramipril/HCTZ C07AA07 - Sotalol  C03DA01 - Spironolactone C03EA01 - Spironolactone/HCTZ C09CA07 - Telmisartan C09DA07 - Telmisartan/HCTZ G04CA03 - Terazosin  C07AA06 - Timolol C07BA06 - Timolol/Thiazides C03CA04 - Torsemide C09AA10 - Trandolapril  C09BB10 - Trandolapril/Verapamil C03DB02 - Triamterene  C03EA01 - Triamteren/HCTZ C09CA03 - Valsartan C09DA03 - Valsartan/HCTZ C08DA01 - Verapamil  C09BB10 - Verapamil/Trandolapril |
| Sickle cell disease  ICD-9 codes: 282.6 ICD-10 codes: D57.1 | Hydroxyurea | L01XX05 – Hydroxycarbamide |
|  | Other hematological agents (B06AX) | B06AX not on the health Canada list of medications |
| **Respiratory** | | |
| Chronic lung disease including COPD, cystic fibrosis, severe asthma  ICD-9 codes: 491-493; 496  ICD-10 codes: J40; J42-J46 | Drugs for obstructive airway diseases (R03) | R03BB05 - Aclidinium bromide R03DA05 – Aminophylline R03BA01 – Beclometasone R03DX10 – Benralizumab R03BA02 – Budesonide  R03AK07 - Budesonide/Formoterol R03AL11 – Budesonide, formoterol, and glycopyrronium  R03BA08 – Ciclesonide R03BC01 – Cromoglicic acid R03DA01 – Diprophylline R03CA02 – Ephedrine  R03AA01 – Epinephrine racemic R03AC04 - Fenoterol  R03CC04 - Fenoterol hydrobromide R03AL01 – Fenoterol and ipratropium  bromide |

| **At-risk medical conditions (ICD-9 and ICD-10 codes)** | **Medicinal product proxy(ies) ATC codes** | |
| --- | --- | --- |
|  |  | R03BA03 – Flunisolide R03BA05 - Fluticasone R03BA09 – Fluticasone furoate  R03AK06 – Fluticasone and salmeterol R03AK10 – Fluticasone and vilanterol R03AL08 - Fluticasone, umeclidinium and vilanterol  R03AC13 - Formoterol furamate R03AL05 – Formoterol/Aclidinium  bromide R03AL07 – Formoterol/  Glycopyrronium bromide R03AK07 – Formoterol/Budesonide R03AK09 – Formoterol/Mometasone R03BB06 - Glycopyrronium bromide R03AL04 – Glycopyrronium bromide and indacaterol  R03AC18 – Indacaterol  R03AK14 – Indacaterol/Mometasone R03AL12 – Indacaterol/Mometasone/ Glycopyrronium  R03BB01 – Ipratropium bromide  R03AK04 - Ipratropium bromide/Albuterol R03AL02 – Ipratropium bromide/  Salbutamol R03AB02 – Isoprenaline R03DX09 – Mepolizumab R03BA07 - Mometasone furoate  R03AK14 - Mometasone/Indacaterol R03AL12 - Mometasone/Indacaterol/ Glycopyrronium  R03DC03 – Montelukast R03BC03 - Nedocromil R03AC19 – Olodaterol R03DX05 – Omalizumab R03AB03 – Orciprenaline R03DA02 - Oxtriphylline  R03DA54 - Oxtriphylline/Guaifenesin R03DA02 – Choline theophyllinate R03AC08 – Pirbuterol  R03AC16 – Procaterol R03DX08 – Reslizumab R03DX07 – Roflumilast R03AC02 - Salbutamol R03CC02 – Salbutamol sulfate  R03AL02 - Salbutamol/Ipratropium bromide R03AC12 – Salmeterol  R03CC03 - Terbutaline sulfate R03AC03 - Terbutaline sulfate  R03DA04 – Theophylline |

| **At-risk medical conditions (ICD-9 and ICD-10 codes)** | **Medicinal product proxy(ies) ATC codes** | |
| --- | --- | --- |
|  |  | R03DB04 – Theophylline and adrenergics R03DA54 – Theophylline, combinations  excl. psycholeptics R03BB04 – Tiotropium bromide R03AL06 – Tiotropium and olodaterol R03BA06 – Triamcinolones  R03BB07 – Umeclidinium bromide R03AL03 – Umeclidinium and vilanterol R03DC01 – Zafirlukast |
|  | Lung surfactants (R07AA) | R07AA01 – Colfosceril palmitate R07AA02 – Natural phospholipids |
|  | Respiratory stimulants (R07AB) | R07AB01 – Doxapram |
| Endocrine | | |
| Type 1 and 2 Diabetes  ICD-9 codes: 250.0-250.9, 271.4  and 790.2  ICD-10 codes: E10-E14 and R73.0 |  | A10BF01 - Acarbose A10BJ04 - Albiglutide A10BH04 - Alogliptin  A10BD13 - Alogliptin/Metformin A10BD09 - Alogliptin/Pioglitazone A10BK02 - Canagliflozin  A10BD16 - Canagliflozin/Metformin A10BB02 - Chlorpropamide A10BK01 - Dapagliflozin  A10BD15 - Dapagliflozin/Metformin A10BD21 - Dapagliflozin/Saxagliptin A10BJ05 - Dulaglutide  A10BK03 - Empagliflozin  A10BD19 - Empagliflozin/Linagliptin A10BD20 - Empagliflozin/Metformin A10BD - Ertugliflozin/Sitagliptin A10BD - Ertugliflozin/Metformin A10BJ01 - Exenatide  A10BB12 - Glimepiride  A10BD04 - Glimepiride/Rosiglitazone H04AA01 - Glucagon  A10BB01- Glyburide A10BH05 - Linagliptin  A10BD11 - Linagliptin/Metformin A10BJ02 - Liraglutide  A10AE56 - Liraglutide/Insulin degludec A10BA02 - Metformin  A10BX03 - Nateglinide A10BG03 - Pioglitazone A10BX02 - Repaglinide A10BG02 - Rosiglitazone  A10BD03 - Rosiglitazone/Metformin A10BH03 - Saxagliptin  A10BD10 - Sazagliptin/Metformin |

| **At-risk medical conditions (ICD-9 and ICD-10 codes)** | **Medicinal product proxy(ies) ATC codes** | |
| --- | --- | --- |
|  |  | A10BH01 - Sitagliptin  A10BD07 - Sitagliptin/Metformin A10BB03 - Tolbutamide A10AB05 - Insulin aspart  A10AD05 - Insulin aspart/Insulin protamine A10AB02 - Insulin beef  A10AE06 - Insulin degludec  A10AE56 - Insulin degludec/Liraglutide A10AE05 - Insulindetemir  A10AE04 - Insulin glargine  A10AE54 - Insulin glargine/Lixisenatide A10AB06 - Insulin glulisine  A10AD01 - Insulin human biosynthetic/ Insulin isophane  A10AC03 - Insulin isophane pork A10AB04 - Insulin lispro  A10AD04 - Insulin lispro/Insulin lispro protamine  A10AC01 - Insulin NPH human DNA origin A10AB03 - Insulin pork  A10AB01 - Insulin semi synthetic human A10AE01 - Insulin zinc human biosynthethic |
| Obesity diagnosis or having a BMI  ≥ 30 kg/m2  ICD-9 codes: 278.0; 649.1; V85.3-V85.4  ICD10-codes: E66; O99.21;Z68.3-Z68.4 | Peripherally acting antiobesity products (A08AB) | A08AB01 - Orlistat |
|  | Centrally acting antiobesity products (A08AA) | A08AA03 - Amfepramone  A08AA62 - Bupropion and Naltrexone A08AA02 - Fenfluramine  A08AA05 - Mazindol A08AA01 - Phentermine A08AA10 - Sibutramine |
| Renal | | |
| **Chronic kidney disease**  ICD-9 codes: 583-586; 592; 593.9  ICD-10 codes: N00-N23 | Erythropoietin (BA3XA01) | B03XA01 - Erythropoietin |
| **Immunological** | | |
| HIV  ICD-9 code: 042 ICD-10 code: B20 | Protease inhibitors (J05AE) | J05AE05 - Amprenavir J05AE08 - Atazanavir J05AE10 - Darunavir J05AE02 - Indinavir J05AE07 - Fosamprenavir J05AE04 - Nelfinavir  J05AE03 - Ritonavir |

| **At-risk medical conditions (ICD-9 and ICD-10 codes)** | **Medicinal product proxy(ies) ATC codes** | |
| --- | --- | --- |
|  |  | J05AE01 - Saquinavir J05AE09 - Tipranavir |
|  | Combinations to treat HIV (J05AR) | J05AR02 - Abacavir and Lamivudine J05AR13 - Abacavir, Dolutegravir, and  Lamivudine  J05AR04 - Abacavir, Lamivudine, and Zidovudine  J05AR20 - Alafenamide, Emtricitabine, and Tenofovir  J05AR15 - Atanazavir and Cobicistat  - Cabotegravir, and Rilpivirine J05AR14 - Cobicistat and Darunavir J05AR18 - Cobicistat, Elvitegravir, Emtricitabine, and Tenofovir J05AR09 - Cobicistat, Elvitegravir, Emtricitabine, and Tenofovir disoproxil  J05AR22 - Cobicistat, Emtricitabine, Darunavir, and Tenofovir alafenamide  J05AR25 - Dolutegravir and Lamivudine J05AR21 - Dolutegravir and Rilpivirine J05AR06 - Efavirenz, Emtricitabine, and  Tenofovir  J05AR19 - Emtricitabine, Rilpivirine, and Tenofovir  J05AR03 - Emtricitabine and Tenofovir disoproxil  J05AR17 - Emtricitabine and Tenofovir alafenamide  J05AR01 - Lamivudine and Zidovudine J05AR05 - Lamivudine, Nevirapine, and  Zidovudine  J05AR10 - Lopinavir and Ritonavir |
|  | NRTI (J05AF) | J05AF06 - Abacavir J05AF08 - Adefovir dipivoxil J05AF02 - Didanosine J05AF09 - Emtricitabine J05AF10 - Entecavir J05AF05 - Lamivudine J05AF04 - Stavudine J05AF11 - Telbivudine  J05AF13 - Tenofovir alafenamide J05AF07 - Tenofovir disoproxil J05AF03 - Zalcitabine  J05AF01 - Zidovudine |
|  | NNRTI (J05AG) | J05AG03 - Efavirenz |

| **At-risk medical conditions (ICD-9 and ICD-10 codes)** | **Medicinal product proxy(ies) ATC codes** | |
| --- | --- | --- |
|  |  | J05AG04 - Etravirine J05AG02 - Delavirine J05AG06 - Doravirine J05AG01 - Nevirapine J05AG05 - Rilpivirine |
| Immunosuppression | Immunosuppressant (L04A) | L04AA24 - Abatacept L04AB04 - Adalimumab L04AA15 - Alefacept L04AA34 - Alemtuzumab L04AC03 - Anakinra L04AA51 - Anifrolumab  L04AA03 - Antilymphocyte immunoglobulin L04AA04 - Antithymocyte immunoglobulin (rabbit)  L04AA32 - Apremilast L04AX01 - Azathioprine L04AA37 - Baricitinib L04AC02 - Basiliximab L04AA26 - Beliumab L04AC12 - Brodalumab L04AC08 - Canakinumab  L04AB05 - Certolizumab pegol L04AD01 - Ciclosporin L04AA40 - Cladribine L04AC01 - Daclizumab L04AX07 - Dimethyl fumarate L04AA25 - Eculizumab L04AA21 - Efalizumab L04AB01 - Etanercept L04AA18 - Everolimus L04AA27 - Fingolimod L04AB06 - Golimumab L04AC16 - Guselkumab L04AB02 - Infliximab  L04AC13 - Ixekizummab L04AA13 - Leflunomide L04AX04 - Lenalidomide L04AA36 - Ocrelizumab L04AA52 - Ofatumumab L04AA38 - Ozanimob L04AX03 - Methotrexate L04AA02 - Muromonab-CD3  L04AA06 - Mycophenolic acid L04AA23 - Natalizumab L04AX05 - Pirfenidone L04AX06 - Pomalidomide L04AA50 - Ponesimod L04AA43 - Ravulizumab  L04AC18 - Rizankizumab |

| **At-risk medical conditions (ICD-9 and ICD-10 codes)** | **Medicinal product proxy(ies) ATC codes** | |
| --- | --- | --- |
|  |  | L04AC14 - Sarilumab L04AC19 - Satralizumab L04AC10 - Secukinumab L04AC11 - Siltuximab L04AA42 - Siponimod L04AA10 - Sirolimus L04AD02 - Tacrolimus L04AX02 - Thalidomide L04AC07 - Tocilizumab L04AA29 - Tofacitinib L04AA31 - Teriflunomide L04AC17 - Tildrakizumab L04AA44 - Upadacitinib L04AC05 - Ustekinumab L04AA33 - Vedolizumab |
|  | Corticosteroids (H02) | H02AB01 - Betamethasone H02AB10 - Cortisone H02AB02 - Dexamethasone H02AA02 - Fludrocortisone H02AB09 - Hydrocortisone  H02AB04 - Methylprednisolone H02AB06 - Prednisolone H02AB07 - Prednisone  H02AB57 - Prednisone, Combinations H02AB08 - Triamcinolone |
| **Cancer** | | |
| Cancer  ICD-9 code: 140-239 ICD-10 code: C00-D49 | Alkylating agents (L01A) | L01AA09 - Bendamustine L01AB01 - Busulfan L01AD01 - Carmustine L01AA02 - Chlorambucil L01AA05 - Chlormethine  L01AA01 - Cyclophosphamide L01AX04 - Dacarazine L01AA06 - Ifosfamide  L01AD02 - Lomustine L01AA03 - Melphalan L01AD04 - Streptozocin L01AX03 - Temozolomide L01AC01 - Thiotepa L01AB02 - Treosulfan |
|  | Antimetabolites (L01B) | L01BC07 - Azacitidine L01BC06 - Capecitabine L01BB04 - Cladribine L01BB06 - Clofarabine L01BC01 - Cytarabine  L01BC08 - Decitabine L01BB05 - Fludarabine |

| **At-risk medical conditions (ICD-9 and ICD-10 codes)** | **Medicinal product proxy(ies) ATC codes** | |
| --- | --- | --- |
|  |  | L01BC02 - Fluorouracil  L01BC52 - Fluorouracil, Combinations L01BC05 - Gemcitabine  L01BB02 - Mercaptopurine L01BA01 - Methotrexate L01BB07 - Nelarabine L01BA04 - Pemetrexed L01BA05 – Pralatrexate L01BA03 - Raltitrexed L01BB03 - Tioguanine  L01BC59 – Trifluridine, Combinations |
|  | Plant alkaloids and other natural products (L01C) | L01CD04 - Cabazitaxel L01CD02 - Docetaxel L01CB01 - Etoposide L01CE02 - Irinotecan L01CD01 – Paclitaxel L01CB02 - Teniposide L01CE01 – Topotecan L01CX01 - Trabectedin L01CA01 – Vinblastine L01CA02 - Vincristine L01CA03 - Vindesine L01CA04 - Vinorelbine |
|  | Cytotoxic antibiotics and related substances (L01D) | L01DC01 – Bleomycin L01DA01 - Dactinomycin L01DB02 - Daunorubicin L01DB01 – Doxorubicin L01DB03 - Epirubicin L01DB06 – Idarubicin L01DC03 – Mitomycin L01DB07 – Mitoxantrone L01DB09 – Valrubicin |
|  | Other antineoplastic agents (L01X) |  |
|  | Hormones and related agents (L02A) | L02AE01 - Buserelin L02AA01 – Diethylstilbestrol L02AE03 - Goserelin L02AE05 - Histrelin  L02AE02 - Leuprorelin L02AB01 – Megestrol L02AE04 - Triptorelin |
|  | Hormone antagonists and related agents (L02B) | L02BX03 – Abiraterone L02BG01 - Aminogluthetimide L02BG03 – Anastrozole L02BB05 - Apalutamide L02BB03 – Bicalutamide L02BB06 - Darolutamide |

| **At-risk medical conditions (ICD-9 and ICD-10 codes)** | **Medicinal product proxy(ies) ATC codes** | |
| --- | --- | --- |
|  |  | L02BX02 – Degarelix L02BB04 - Enzalutamide L02BG06 – Exemestane L02BB01 – Flutamide L02BG02 - Formestane L02BA03 – Fulvestrant L02BG04 – Letrozole L02BB02 - Nilutamide L02BA01 – Tamoxifen |
|  | Immunostimulants (L03)  Remove L03AX03 – BCG  vaccine And  L03AX12 – Melanoma vaccine | L03AC01 – Aldesleukin L03AA12 - Ancestim L03AA02 - Filgrastim  L03AX13 – Glatiramer acetate L03AB09 – Interferon Alfacon-1 L03AB04 – Interferon Alfa-2A L03AB05 – Interferon Alfa-2B L03AB07 – Interferon Beta-1A L03AB08 – Interferon Beta-1B L03AB06 – Interferon Alfa-N1 L03AA13 – Pegfilgrastim L03AB11 – Peginterferon Alfa-2A  L03AB60 – Peginterferon Alfa-2A,  Combinations L03AB10 – Peginterferon Alfa-2B L03AB60 – Peginterferon Alfa-2B,  Combinations L03AB13 – Peginterferon Beta-1A L03AX16 - Plerixafor |
|  | Immunosuppressants (L04) | L04AA24 - Abatacept L04AB04 – Adalimumab L04AA15 – Alefacept L04AA34 - Alemtuzumab L04AC03 – Anakinra L04AA51 – Anifrolumab  L04AA03 – Antilymphocyte immunoglobulin (horse)  L04AA04 – Antithymocyte immunoglobulin (rabbit)  L04AA32 - Apremilast L04AX01 – Azathioprine L04AA37 – Baricitinib L04AC02 - Basiliximab L04AA26 – Belimumab L04AC12 - Brodalumab L04AC08 - Canakinumab  L04AB05 – Certolizumab pegol L04AD01 – Ciclosporin L04AA40 – Cladribine |

| **At-risk medical conditions (ICD-9 and ICD-10 codes)** | **Medicinal product proxy(ies) ATC codes** | |
| --- | --- | --- |
|  |  | L04AC01 - Daclizumab L04AX07 – Dimethyl fumarate L04AA25 - Eculizumab L04AA21 - Efalizumab L04AB01 – Etanercept L04AA18 - Everolimus L04AA27 – Fingolimod L04AB06 – Golimumab L04AC16 - Guselkumab L04AB02 – Infliximab  L04AC13 - Ixekizumab L04AA13 – Leflunomide L04AX04 – Lenalidomide L04AX03 – Methotrexate L04AA02 – Muromonab-CD3 L04AA06 – Mycophenolic acid L04AA23 - Natalizumab L04AA36 - Ocrelizumab L04AA52 – Ofatumumab L04AA38 - Ozanimob  L04AX05 – Pirfenidone L04AX06 – Pomalidomide L04AA50 – Ponesimod L04AA43 - Ravulizumab L04AC18 - Risankizumab L04AC14 - Sarilumab L04AC19 - Satralizumab L04AC10 – Secukinumab L04AC11 - Siltuximab L04AA42 - Siponimod L04AA10 - Sirolimus L04AD02 – Tacrolimus L04AA31 – Teriflunomide L04AX02 - Thalidomide L04AC17 - Tildrakizumab L04AC07 – Tocilizumab L04AA29 - Tofacitinib L04AA44 – Upadacitinib L04AC05 - Ustekinumab L04AA33 - Vedolizumab |
| **Mental disorders** | | |
| **Mood and anxiety disorders**  **Bipolar:**  ICD-9 codes: 296.0-296.1; 296.4-  296.8  ICD-10 codes: F30; F31; F34.0  **Depression :** |  | N05BA12 - Alprazolam N06AA09 - Amitriptyline N06AA17 - Amoxapine N06AX12 - Bupropion N05BE01 - Buspirone N06AB04 - Citalopram  N06AA04 - Clomipramine N03AE01 - Clonazepam |

| **At-risk medical conditions (ICD-9 and ICD-10 codes)** | **Medicinal product proxy(ies) ATC codes** | |
| --- | --- | --- |
| ICD-9 codes: 296.2; 296.3; 300.4;  311  ICD-10 codes: F32; F33; F34.1, F38.1  **Other :**  ICD-9 code: 296.9  ICD-10 codes: F34.8; F34.9; F38.0; F38.8; F39  **Anxiety :**  ICD-9 codes: 300.0; 300.2; 300.3;  309.8  ICD-10 codes: F40; F41; F42; F93.0- F93.2  **Acute stress :**  ICD-9 code: 308.3  ICD-10 codes: F43.0; F43.1; F43.8; F43.9 |  | N06AA01 - Desipramine N06AX23 - Desvenlafaxine N05BA01 - Diazepam N06AA12 - Doxepin N06AX21 - Duloxetine N06AB10 - Escitalopram N06AB03 - Fluoxetine N06AB08 - Fluvoxamine N06AA02 - Imipramine N06AF01 - Isocarboxazid N06AX28 - Levomilnacipran N05BA06 - Lorazepam N06AX02 – L-tryptophane N06AA21 - Maprotiline N06AX17 - Milnacipran N06AX11 - Mirtazapine N06AG02 - Moclobemide N06AX06 - Nefazodone N06AA10 - Nortriptyline N06AB05 - Paroxetine N06AF03 – Phenelzine N06AA11 - Protriptyline N06AX18 - Reboxetine N04BD01 - Selegiline N06AB06 - Sertraline N06AF04 - Tranylcypromine N06AX05 - Trazodone N06AA06 - Trimipramine N06AX16 - Venlafaxine N06AX24 - Vilazodone N06AX26 – Vortioxetine |
| **Other mental disorders Organic disorders:**  **Senile and pre-senile psychotic conditions**  ICD-9 codes: 290.0-290.9 ICD-10 codes: F00-F09; G30  **Transient organic psychotic conditions**  ICD-9 codes: 293.0; 293.1; 293.8;  293.9  ICD-10 codes: None  **Schizoprenic/psychotic disorders: Schizoprenia**  ICD-9 codes: 295.0-295.9 | Antipsychotics 1st and 2nd generations | N05AX12 - Aripiprazole N05AH05 - Asenapine N05AA01 - Chlorpromazine N05AH02 - Clozapine N01AX01 - Droperidol N05AD08 - Droperidol N05AB02 - Fluphenazine N05AD01 - Haloperidol N05AH01 - Loxapine N05AE05 - Lurasidone N05AH03 - Olanzapine N05AX13 - Paliperidone N05AB03 - Perphenazine N05AH04 - Quetiapine N05AX08 - Risperidone N05AC02 - Thioridazine  N05AF04 - Thothixene N05AB06 - Trifluoperazine |

| **At-risk medical conditions (ICD-9 and ICD-10 codes)** | **Medicinal product proxy(ies) ATC codes** | |
| --- | --- | --- |
| ICD-10 codes: F20-F29 |  | N05AE04 - Ziprasidone |
| **Psychotic** |  |  |
| ICD-9 codes: 298.8-298.9 |  |  |
| ICD-10 codes: None |  |  |
| **Paranoia** |  |  |
| ICD-9 codes: 297.1-297.3 |  |  |
| ICD-10 codes: None |  |  |
| **Personality disorders :** |  |  |
| ICD-9 codes: 301.0-301.9 |  |  |
| ICD-10 codes: F60; F61; F62; F69 |  |  |
| **Other disorders :** |  |  |
| **Adjustment disorders** |  |  |
| ICD-9 codes: 309.0-309.4; 309.8- |  |  |
| 309.9 |  |  |
| ICD-10 codes: F43.2; F99 |  |  |
| **Sexual disorders** |  |  |
| ICD-9 codes: 302.0-302.9 |  |  |
| ICD-10 codes: F52; F64; F65; F66 |  |  |
| **Delusional disorders** |  |  |
| ICD-9 codes: 297.0-297.3; 297.8- |  |  |
| 297.9 |  |  |
| ICD-10 codes: None |  |  |
| **Disturbance of conduct not** |  |  |
| **otherwise specified** |  |  |
| ICD-9 codes: 312.0-312.4; 312.8- |  |  |
| 312.9 |  |  |
| ICD-10 codes: F63; F91; F92; F95 |  |  |
| **Non-organic psychoses** |  |  |
| ICD-9 codes: 298.0-298.4 |  |  |
| ICD-10 codes: None |  |  |
| **All other psychiatric disorders** |  |  |
| ICD-9 codes: 299.0; 299.1; 299.8; |  |  |
| 299.9; 300.1; 300.5-300.9; 307.0- |  |  |
| 307.7; 307.9; 308.0-308.2; 308.9, |  |  |
| 310, 313.0-313.3; 313.8; 313.9; |  |  |
| 314.0-314.2; 314.8; 314.9; 315.0- |  |  |
| 315.5; 315.9; 316; 317; 318.0-318.2; |  |  |
| 319 |  |  |
| ICD-10 codes: F44; F48; F50; F51; |  |  |
| F53; F54; F68; F70-F73; F84; F98; |  |  |

| **At-risk medical conditions (ICD-9 and ICD-10 codes)** | **Medicinal product proxy(ies) ATC codes** | |
| --- | --- | --- |
| F79; F78; F80-F83; F88; F89; F90; F93.3-F93.9, F94 |  |  |
| **Common rheumatic diseases** | | |
| **Osteoarthritis and allied** | Disease-modifying | L04AA32 - Apremilast |
| **disorders:** | antirheumatic drugs | L04AX01 - Azathioprine |
| ICD-9 code: 715 | (DMARDs) | L04AA37 - Baricitinib |
| ICD-10 codes: M15.0; M15.9; |  | L01AA01 - Cyclophosphamide |
| M15.1; M15.2; M19.9; M19.019; |  | L04AD01 - Cyclosporine |
| M19.029; M19.039; M19.049; |  | P01BA02 - Hydroxychloroquine |
| M16.10; M17.10; M19.079; M19.91; |  | L04AA13 - Leflunomide |
| M19.93; M19.219; M19.229; |  | L04AX03 - Methotrexate |
| M19.239; M19.249; M16.7; M17.5; |  | L04AA06 – Mycophenolate mofetil |
| M19.279; M19.93; M19.90; M18.9; |  | A07EC01 - Sulfasalazine |
| M16.9; M17.9; M15.8; M15.3; |  | L04AA29 - Tofacitinib |
| M15.9; M18.9 |  |  |
| **Rheumatoid arthritis (RA)** |  |  |
| ICD-9 code: 714 |  |  |
| ICD-10 codes: M05; M06 |  |  |
| **Systemic lupus erythematosus** |  |  |
| **(SLE)** |  |  |
| ICD-9 codes: 710.0; 695.4 |  |  |
| ICD-10 codes: M32.10; L930; L932 |  |  |
| **Ankylosing spondylitis** |  |  |
| ICD-9 codes: 720-721 |  |  |
| ICD-10 codes: M45; M47 |  |  |
| **Psoriatic arthritis** |  |  |
| ICD-9 code: 696.0 |  |  |
| ICD-10 code: L40.5 |  |  |
| **Sjὅgren syndrome** |  |  |
| ICD-9 code: 710.2 |  |  |
| ICD-10 code: M35.0 |  |  |
| **Gout and crystal arthropathies** |  |  |
| ICD-9 codes: 274; 272 |  |  |
| ICD-10 codes: M10**;** M11 |  |  |
| **Scleroderma/systemic sclerosis** |  |  |
| ICD-9 code: 710.1 |  |  |
| ICD-10 code: M34.0 |  |  |
| **Polymyalgia rheumatica** |  |  |
| ICD-9 code: 725 |  |  |
| ICD-10 code: M35.3 |  |  |

| **At-risk medical conditions (ICD-9 and ICD-10 codes)** | **Medicinal product proxy(ies) ATC codes** | |
| --- | --- | --- |
|  |  |  |
| **Other covariates** | | |
| **Tobacco dependence**  ICD-9 codes: 305.0; 305.1 and  649.0  ICD-10 codes: F17; O99.33; Z71.6,  and Z72.0 | Nicotine replacement  CCI codes | N07BA01 – Nicotine N07BA03 – Varenicline  7SP10VK - Counsel promote health smoking 5AD14BK |
| **Alcohol dependence** | Medications Used to | N07BB01 – Disulfiram |
| ICD-9 codes: 303; 305; 357.5; | Treat Alcohol | N07BB03 – Acamprosate |
| 425.5; 535.3; 571.0-571.4 and 980 | Withdrawal Symptoms | N07BB04 - Naltrexone |
| ICD-10 codes: F10; G31.2; G62.1; |  |  |
| I42.6; K29.2; K70; K85.2; K86.0 and |  |  |
| O99.31 | CCI codes | 7SP60VA - Educate promote health alcohol |
|  |  | (mis)use |
|  |  | 5AD14BK - Antepartum counsel, lifestyle |
|  |  | adjust |
